# Digital Printing of Photoreversible Protective Films

**DOI:** 10.1002/marc.70408

**Published:** 2026-08-17

**Authors:** Morris Wolf, Alper Balkan, René Schneider, Stefan Mommer, Yves Leterrier, Mark W. Tibbitt

**Affiliations:** ^1^ Macromolecular Engineering Laboratory ETH Zurich Zürich Switzerland; ^2^ Laboratory for Processing of Advanced Composites (LPAC) École Polytechnique Fédérale de Lausanne (EPFL) Lausanne Switzerland; ^3^ Laboratory For Functional Polymers Swiss Federal Laboratories for Materials Science and Technology (Empa) Switzerland

**Keywords:** adhesion, coatings, photoreversible polymers, printing

## Abstract

Temporary protective coatings are widely used in modern production processes. Yet, while highly desirable, their high‐resolution printing and stimuli‐responsive removal remain challenging. To address this need, we introduce acrylate‐based photopolymerized and photoreversible protective films that can be applied using aerosol jet printing. The acrylate films were formulated using aqueous solutions of a photocleavable diacrylate cross‐linker and acrylated macromers. To form films, the precursor solution was cured with 405 nm light for 120 s. The films were subsequently removed via photodegradation upon exposure to 365 nm light within minutes to hours depending on film thickness and light intensity. The addition of a polyethyleneimine‐based adhesion promoter, Lupasol WF, led to significant improvement of adhesion to industry‐relevant surfaces. Quantification using a rheometer in extensional mode showed a fracture energy of the films 3.25x higher than without the adhesion promoter. The protective films were applied using aerosol jet printing, enabling a feature resolution below 30 µm and a spatially selective treatment of metallic surfaces with Paris oxide. These results demonstrate how photopolymerized and photoreversible polymer networks enable temporary protective coatings in industry‐relevant applications.

## Graphical abstract

A photopolymerized and photoreversible temporary protective film based on an ortho‐nitrobenzyl crosslinker is presented that can be applied using aerosol jet printing. To improve adhesion to industry‐relevant surfaces, a polyethyleneimine‐based adhesion promoter was added to the aqueous formulation of diacrylate cross‐linker and acrylated macromers. The films enabled spatially selective treatment of metallic surfaces.

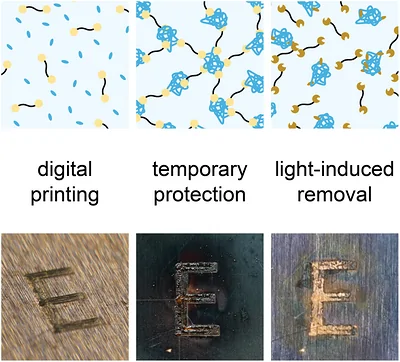

## 1 Introduction

Temporary protective films are useful in many industrial applications, including precise masking to limit the treated area in various surface finishing processes or fully covering parts to protect them during subsequent steps of production. In some manufacturing processes, such as micro‐ and nano‐electromechanical systems (MEMS/NEMS) manufacturing, protective coatings are routinely integrated in specialized processes using positive and negative photoresists [1, 2, 3, 4, 5]. These approaches allow high‐resolution surface modification but are often limited to established processes, such as spin coating developed for use on standardized silica wafers, leading to deposition across the whole, flat surface [6] without selectivity [7]. Current needs beyond uniform deposition on silicon wafers include stretchable electronics, geometrically complex substrates, or new coating materials, increasing the interest in novel application techniques of films [8, 9, 10, 11, 12]. For example, in the watch industry, many parts are different in size, shape, and material, motivating the need for temporary protective films that can be applied to arbitrary geometries at high‐resolution using advanced manufacturing techniques. Reversible coatings that can be applied via digital printing technologies would complement current coating approaches based on manual application and removal. In addition, printable and reversible coating technologies would widen the possibilities for surface treatments in terms of resolution, substrate geometry, and removal without the need for mechanical manipulation.

One strategy to engineer advanced coating materials is through the design of polymer networks, as they offer a large variety of chemistries, including a wide range of possible stimuli to initiate changes in mechanical properties or reversibility, e.g., via temperature [13, 14], pH [15, 16, 17, 18], chemical triggers [19, 20], or light [21, 22]. Photodegradation of films using light offers specific advantages: it allows for precise spatiotemporal control, is contactless, and can be integrated easily in standard industrial processes. Several molecular approaches have been integrated into polymer networks to achieve photodegradation [23, 24, 25]. For example, coumarin has been studied for its ability to undergo direct cleavage, for example releasing biomolecules [26]. In addition, photocycloaddition of coumarins forms cyclobutane rings through dimerization (λ > 300 nm) that can be cleaved upon irradiation at shorter wavelengths (λ < 300 nm) [27]. However, in this case, full cleavage can be hard to achieve as the processes of photocycloaddition and photocleavage are in dynamic equilibrium [28]. Another well‐studied class of photoreversible chemistries are azobenzenes, which have been used as reversible photo switches [29, 30, 31] as they undergo reversible trans–cis isomerization initiated by light. Hexaarylbiimidazoles have the capability to cleave under irradiation—a process that is reversible in the absence of light [32]. While particularly useful for self‐healing materials [33], hexaarylbiimidazoles are less suitable to design fully cleavable networks due to the dark‐state reversibility.

One prominent strategy to design photodegradable materials with complete and permanent cleavage is the incorporation of ortho‐nitrobenzyl (oNB) moieties into the network structure [25, 34], due to their ability to undergo irreversible cleavage in response to UV‐light (300–365 nm) [35, 36, 37]. Many photodegradable networks based on oNBs have been introduced, including those based on ether, methacrylate, epoxy, or alkene functionalized molecules [34, 36], that have also been used for the formation of photoreactive films [38, 39]. Such films were designed primarily as photoresists for application on broad surfaces, for example via spin coating [39, 40]. However, these materials are not optimized for local deposition in combination with local removal—a combination that would allow printing of protective coatings with fine features which can be selectively removed through light.

Networks formed via small molecule precursors have a high density of photon‐absorbing oNBs, often resulting in slow and energy‐intensive cleavage [38, 39]. Enhanced degradation has been achieved by designing networks with lower oNB‐density through the incorporation of longer polymer chains between the cleavable units while maintaining the ability to fully degrade. One such approach is a cross‐linker based on poly(ethylene glycol) (PEG) functionalized on both sides with acrylated oNB, as photodegradable acrylate (PDA) [41]. The resulting PEGdiPDA cross‐linker results in fully cleavable polymer networks that were originally developed as photodegradable hydrogels for biomedical applications [42, 43, 44, 45]. The efficient degradation kinetics and complete reversibility of PEGdiPDA networks also make them attractive for industrial applications.

In this work, we adapted PEGdiPDA networks to design water‐based, low‐viscosity formulations amenable to digital printing technologies as a strategy to engineer printable and reversible coating materials. Aqueous formulations are not only suitable to achieve low viscosity, but they are also optimized for sustainable solutions in industrial applications, an increasingly prominent focus for the translation to applications [46]. We demonstrated that polymer films based on oNB‐moieties could be applied, photocured, and photodegraded with light sources and time scales relevant to industrial processes. To increase the surface adhesion of the materials, we introduced polyethyleneimines (Lupasol WF) into the formulation, as they are known adhesion promoters for a range of surfaces [47, 48]. The addition of polyethyleneimines significantly increased the adhesion of the material in immersion‐based adhesion tests on metal, as well as in rheometer‐based pull‐out tests for in situ cured soft materials. This rendered the coatings suitable as protective films in downstream surface treatment processes. The polyethyleneimine‐containing films retained their ability to photocure and photodegrade and locally protected metal surfaces during Paris oxide blackening. To apply the coatings at high‐resolution, we tailored the formulation for aerosol jet printing (AJP), and the temporary protective function of the film was demonstrated through a staining process on copper. Overall, this approach demonstrates how photocurable and photodegradable networks can be used as printable and reversible protective coatings in industry‐relevant applications.

## 2 Materials and Methods

### 2.1 Materials

PEGdiPDA [41] and Lithium phenyl‐2,4,6‐trimethylbenzoylphosphinate (LAP) [49, 50] were synthesized using established protocols from literature. For the PEGdiPDA synthesis, the commercially available photolinker 4‐(4‐(1‐Hydroxyethyl)‐2‐methoxy‐5‐nitrophenoxy)butanoic acid (containing the oNB‐group) was functionalized with acryloyl chloride and coupled to both ends of a NH2‐PEG‐NH2 (MW = 3500) molecule. To confirm the synthesis of PEGdiPDA, ^1^H‐NMR was recorded in (CD_3_)_2_SO on a Bruker Avance III 400 NMR spectrometer (Bruker BioSpin Corp., Billerica, MA, USA). Spectroscopic data was consistent with those reported in the literature (Figure S1). In addition, the resulting molecule was analyzed using GPC, confirming successful functionalization to obtain the PEGdiPDA with a small residual fraction of monofunctional product (Figures S2 and S3). PEG‐Acrylate (PEGA) (*M_n_* ∼480 g mol^−1^) was purchased through Sigma–Aldrich and used as sold. Lupasol WF (*M_n_* ∼25 000 g mol^−1^) was purchased from BASF and serves as a polyethyleneimine‐based adhesion promoter. MilliQ‐water (dH_2_O) was used as the aqueous solvent in all experiments.

### 2.2 Gel Permeation Chromatography (GPC)

SEC analyses were carried out on an OMNISEC Resolve system, equipped with an OMNISEC Reveal triple detector (refractive index, light scattering, and viscosity). Milli‐Q water was used as an eluent and contained 0.1 M of sodium nitrate and 0.02% (w/v) sodium azide, which was filtered through a 0.2 µm Nylon filter before use. One guard column (8 × 50 mm, AGuard, Viscotek) and two A6000M general mixed gel columns (8 × 300 mm) were applied at a flow rate of 0.7 mL·min^−1^ at 25°C, each sample was injected twice. The incremental refractive index value was set to dn/dc = 0.132, as reported for PEO in water. For sample preparation, polymerized samples were dissolved in the carrier phase at a concentration of 0.25–2 mg·mL^−1^ at ambient conditions. The system runs on triple detection mode, and the results were analyzed and exported using the OMNISEC Software (Version 11.32, Malvern Panalytical).

### 2.3 Preparation of the Film

Stock solutions of all ingredients were prepared in dH_2_O, PEGdiPDA at 25 wt.%, PEGA at 40 wt.%, Lupasol WF at 10 wt.%, and LAP at 5 wt.%. The precursor solutions were prepared with a constant 15 wt.% of PEG‐based monomers. For 100 µL of material, this resulted in 32.8 µL of PEGdiPDA stock for 8.2 wt.%, 17.0 µL PEGA for 6.8 wt.%, and 10 µL LAP stock for 0.5 wt.%. The remaining volume of 40.2 µL was used to add variable amounts of Lupasol WF with 0 wt.% as the lowest (40.2 µL of water added) and 2 wt.% as the highest. The intensity of light for curing and cleaving was calibrated using a Thorlabs S120VC light sensor with a Thorlabs PM100D optical power meter if not stated differently.

### 2.4 Shear Rheometry of Polymer Films

An Anton Paar MCR 502 Rheometer with a parallel plate of 8 mm diameter (Anton Paar PP08) and a quartz bottom plate with attached liquid light guide (Thorlabs LLG5) was used. For in situ curing and cleaving, the solutions were prepared as described above and loaded with a gap size of 50 µm. Over the course of the measurement, strain amplitude and frequency were kept constant at 2% and 0.5 Hz, respectively. At t = 1 min, 405 nm light from a Thorlabs M405L4‐C1 with driver LEDD1B at an intensity of 5.7 mW cm^−2^ was turned on and then turned off at t = 3 min. Between t = 5 min and t = 6 min, water was added around the sample to prevent drying. The lamp was exchanged for a Thorlabs LED4D007 with driver DC4104 to provide light at 365 nm at an intensity of 9.6 ± 0.7 mW cm^−2^. This lamp was turned on at t = 15 min and not switched off until the end of the measurement. All samples were measured for at least 25 min.

### 2.5 Characterization With UV–vis

For formulations with 0% and 2% Lupasol, 5 µL of solution were prepared and diluted with 400 µL water (1:80 dilution to prevent saturation in measurements). For each formulation, three samples of 100 µL were pipetted in 96‐well plates, as well as 100 µL of deionized water in a separate well as a blank sample. The UV–vis spectrum of each sample was measured with a Hidex plate reader. The samples were irradiated first under 405 nm light (Thorlabs M405L4‐C1 with driver LEDD1B) at 10 mW cm^−2^ for 2 min, measured, then irradiated under 365 nm light (Thorlabs M365L2‐C1 with driver LEDD1B) at 10 mW cm^−2^ for 30 min and measured again.

### 2.6 Characterization With FT‐IR

For formulations with 0% and 2% Lupasol, 3 samples of 4 µL solution were prepared. In Eppendorf tubes, 1 sample was irradiated for 3 min under 405 nm light (Thorlabs M405L4‐C1 with driver LEDD1B) at 14.2 mW cm^−2^ and one sample was irradiated under 405 nm light (Thorlabs M405L4‐C1 with driver LEDD1B) at 14.2 mW cm^−2^ for 3 min, measured, then irradiated under 365 nm light (Thorlabs M365L2‐C1 with driver LEDD1B) at 9.9 mW cm^−2^ for 30 min. Of each sample, 3.5 µL were measured three times on a Perkin Elmer Spectrum Two FT‐IR with 32 scans.

### 2.7 Preparation of Surfaces

Stainless steel (304S15) was purchased from RS Pro (304S15) as a 300 × 300 × 1 mm sheet, and copper was purchased from Reely (297844) as a 400 × 200 × 1 mm sheet. Both were cut into suitable pieces. The surface was not further modified. Before use, the metal parts were rinsed with acetone and then air dried.

### 2.8 Adhesion Testing

The adhesion of the cured films on various surfaces was tested using two different methods, first via observations during immersion in water, and second using an in situ curing and pull‐out test in a rheometer. To test the material's adhesion during immersion, 10 drops of 2 µL were pipetted on stainless steel and cured with 405 nm light at 7.1 mW cm^−2^ for 10 min. The prepared plates were immersed in deionized water at room‐temperature. After 0, 1, 5, 10, 15, 20, 30, 45, 60, 75, 90, 105, 120, 150, 180 and 240 min, the number of sticking dots was counted. This experiment was repeated three times for each formulation.

A novel in situ curing and pull‐out test in a rheometer was developed for quantitative assessment of the fracture energy, with the benefit of enabling testing of small sample volumes. In general, the testing of hydrogel adhesion has been conventionally reported using methods such as lap shear and peel tests [51, 52, 53]. A single lap shear test can require millimeters of material thickness [54], likewise for peel tests, as well as the tested area reaching centimeters [55]. Therefore, adhesion test miniaturization is desirable, which was previously realized for tackiness testing of composite prepregs by a rheometer [56]. This way, higher thickness precision and less material requirement were achieved. In situ curing and pull‐out tests were performed using a rheometer (Anton Paar MCR 302e, Austria) with a photo‐curing accessory (L‐PP/GL/CTD) to analyze the adhesion of photocured films with and without Lupasol WF. The analysis included the strain at break, the maximum pull force, the fracture energy, and the failure mode. A parallel plate geometry consisting of an aluminum (EN AW‐6082) top plate of 8 mm diameter and a quartz bottom plate was used at a gap of 20 µm. The 8 mm diameter of the top plate ensured that the required pull force remained below the maximum force limit of the instrument (∼16.2 N). The OmniCure S2000 UV1‐Standard Lamp (Exfo, Canada) light source was filtered with a 400–500 nm filter (Excelitas, Canada) and calibrated to 8 mW cm^−2^ with the blue light radiometer (400–470 nm) ACCU‐CAL‐50‐V (Dymax, USA), leading to a comparable 405 nm intensity as in other experiments. After a 1 min wait time on the rheometer to ensure sample stability, the formulations were photocured for 2 min and 40 s. The photocuring was followed by 20 s wait time for further stability monitoring. The top plate was then pulled out at a rate of 5 µm s^−1^ until sample break. Normal force and gap were recorded throughout the tests. The gap at the beginning of the pull‐out test (after photo‐curing) was normalized for force–displacement, and therefore stress–strain and fracture energy analysis.

### 2.9 Printing

The 15 wt.% formulations with 0% and 2% Lupasol as described above were diluted for printing on a NanoJet Desktop aerosol jet printer (IDS, USA) to 1.67 wt.% in a way that the final solution had a solvent content of 85 vol.% dH_2_O, 13 vol.% isopropanol, and 2 vol.% n‐butanol, adapting a solvent mixture from literature [57]. In this calculation, the polymer components were included in the dH_2_O volume.

Lines were printed at a speed of 180 mm min^−1^ and gas flows of 4 standard cubic centimeters per minute (sccm) and 70 sccm for the aerosol and the sheath gas, respectively. For the area patches (1 × 2 mm, filled with printed lines with distance 0.01 mm) gas flows were adjusted to 5.5 and 65 sccm for the aerosol and the sheath gas, respectively. All prints were performed with a plastic nozzle with 250 µm inner diameter. Printing was conducted on stainless steel, copper, and glass substrates at 70°C substrate table temperature. The coatings were cured with 405 nm light at 5.7 mW cm^−2^ for 2 min (Thorlabs M405L4‐C1 with driver LEDD1B).

### 2.10 3‐D Imaging of Printed Films

To evaluate the topography, a nondestructive, confocal 3D Optical Surface Metrology System (DCM8, Leica Microsystems AG, Switzerland), equipped with Leica EPI 50x and EPI 150x objectives, was used.

### 2.11 Testing of Printed Samples

A letter “E,” 1.25 × 2.5 mm in size, was printed on copper with the parameters described above. To color the surface while protected by the printed coating, Paris Oxide (Wieland) was dropped onto the surface twice, allowing the volatile solution to fully evaporate in between (approximately 1 min). The sample was irradiated with 365 nm light (Thorlabs M365L2‐C1 with driver DC4104) at 14.2 mW cm^−2^ for 3.5 h and then immersed for 6 min in deionized water at room‐temperature. The plate was irradiated again for 10 min under the same conditions and immersed in water for 1 min. The steps were documented using a Sony alpha7 RII camera as well as a custom microscope from Thorlabs with uEye software.

## 3 Results and Discussion

### 3.1 Formulation of Photocurable and Photodegradable Films

To engineer coating materials that can be prepared as low‐viscosity solutions, photocured, and photodegraded (Figure 1A), we set out to design photoactivatable resins based on an established photodegradable acrylate‐based cross‐linker, PEGdiPDA (Figure 1B) [41]. We co‐formulated the cross‐linker with a monoacrylated PEG (PEGA) to increase the length of acrylate kinetic chains in the network and, thus, enable robust film formation [58]. Importantly, both monomers are water soluble, allowing preparation of low‐viscosity precursor solutions in aqueous formulations. Lithium phenyl‐2,4,6‐trimethylbenzoylphosphinate (LAP) was added as a photoinitiator. Upon irradiation (λ_1_ = 405 nm), radicals were generated, initiating the formation of acrylate kinetic chains that become cross‐linked by PEGdiPDA to form a stable network (Figure 1C). Owing to the inclusion of oNB in the cross‐linker, the PDA can cleave upon irradiation (λ_2_ = 365 nm), allowing the network cross‐links to break and the network to degrade (Figure 1C). With this basic principle, photocurable and photodegradable materials with different properties can be designed, either on a molecular level by changing the length of the PEG chains or by tuning the formulation through the choice of solvent and total polymer content. As many printing technologies require low viscosity formulations [59] and industrial applications favor sustainable solvents with low toxicity, the formulations used here were water‐based.

**FIGURE 1:**
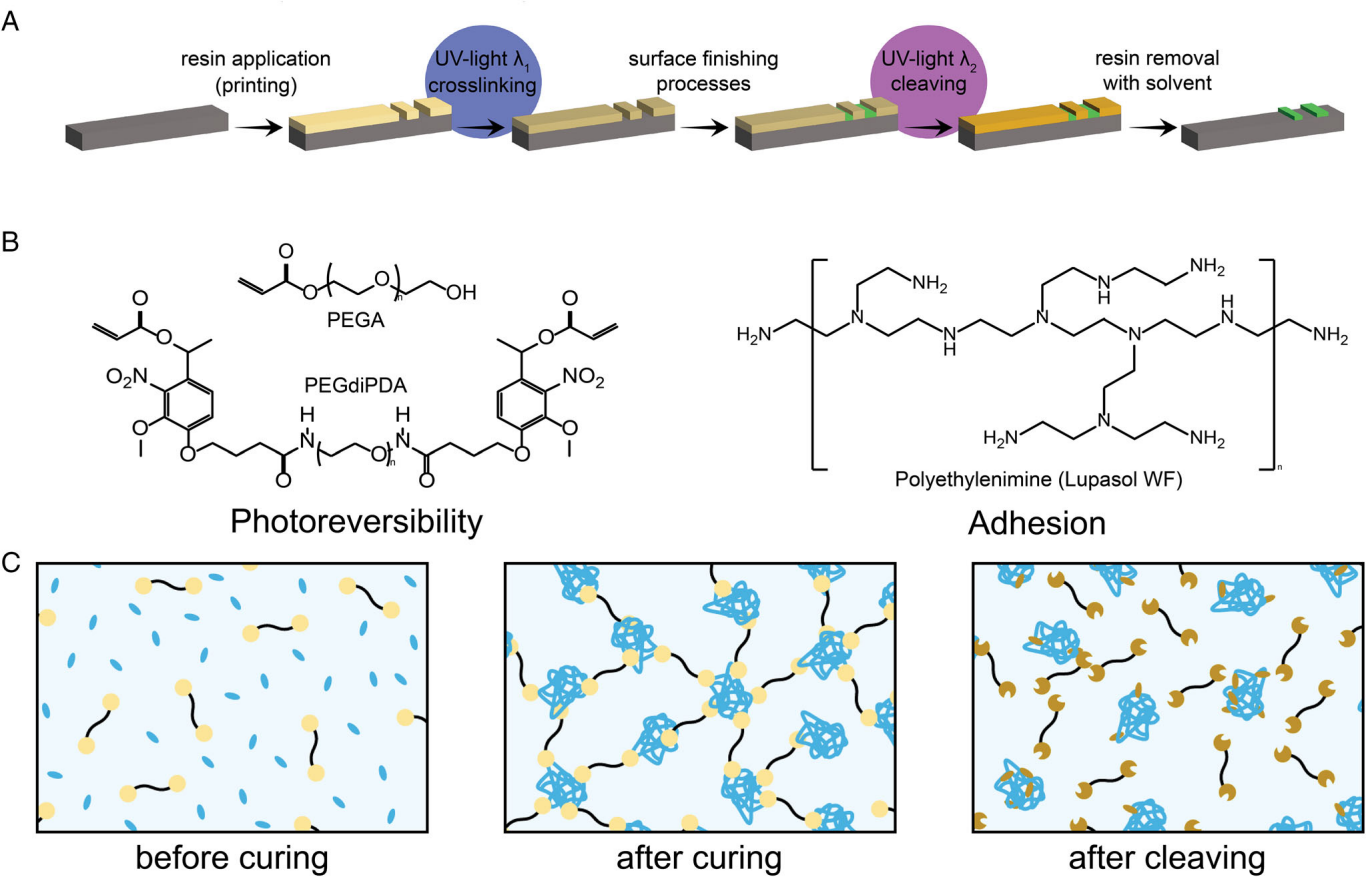
Photocurable and photodegradable protective coatings. (A) The proposed process using a photodegradable film includes application through printing followed by cross‐linking and treatment of the unprotected surface. Subsequently, the film is cleaved with another wavelength of light, allowing the film to be washed away. (B) The two macromers, PEGA and PEGdiPDA, form the photodegradable network, while the long chains of Lupasol WF improve the adhesive properties. (C) Schematic illustration of the basic network architecture with PEGA in blue and PEGdiPDA as black PEG‐chains with photolabile units on both ends in yellow. During curing, the PEGA forms long chains that are connected through PEGdiPDA. Upon cleaving irradiation, the PDA breaks and disconnects the PEGA‐chains. The long Lupasol WF chains that are not bound into the network are omitted for clarity.

As a base formulation, a total polymer content of 15 wt.% in dH_2_O was chosen, composed of 8.2 wt.% PEGA and 6.8 wt.% PEGdiPDA. In addition, 0.5 wt.% LAP was included as a photoinitiator for the polymerization. On the rheometer, the base formulation was dropped on the rheometer plate and cured rapidly within 2 min and cleaved fully in <10 min (Figure 2A). The cleavage followed a characteristic exponential decrease in the storage modulus, indicating pseudo‐first‐order kinetics driven by photoactivation and bond breakage in the oNB moieties [45, 58, 60]. With a gap size of 50 µm, the material is thicker than most printed coatings might be; nevertheless, as this experiment was conducted in the swollen state, evaporation would lead to a much thinner film. These irradiation experiments were conducted with an irradiation source that is relatively low intensity relative to industrial settings. Thus, the effective cleavage time can, in principle, be shortened by a process‐specific set up with stronger lamps, as the cleaving speed of oNBs is proportional to the influx of photons absorbed [61].

**FIGURE 2:**
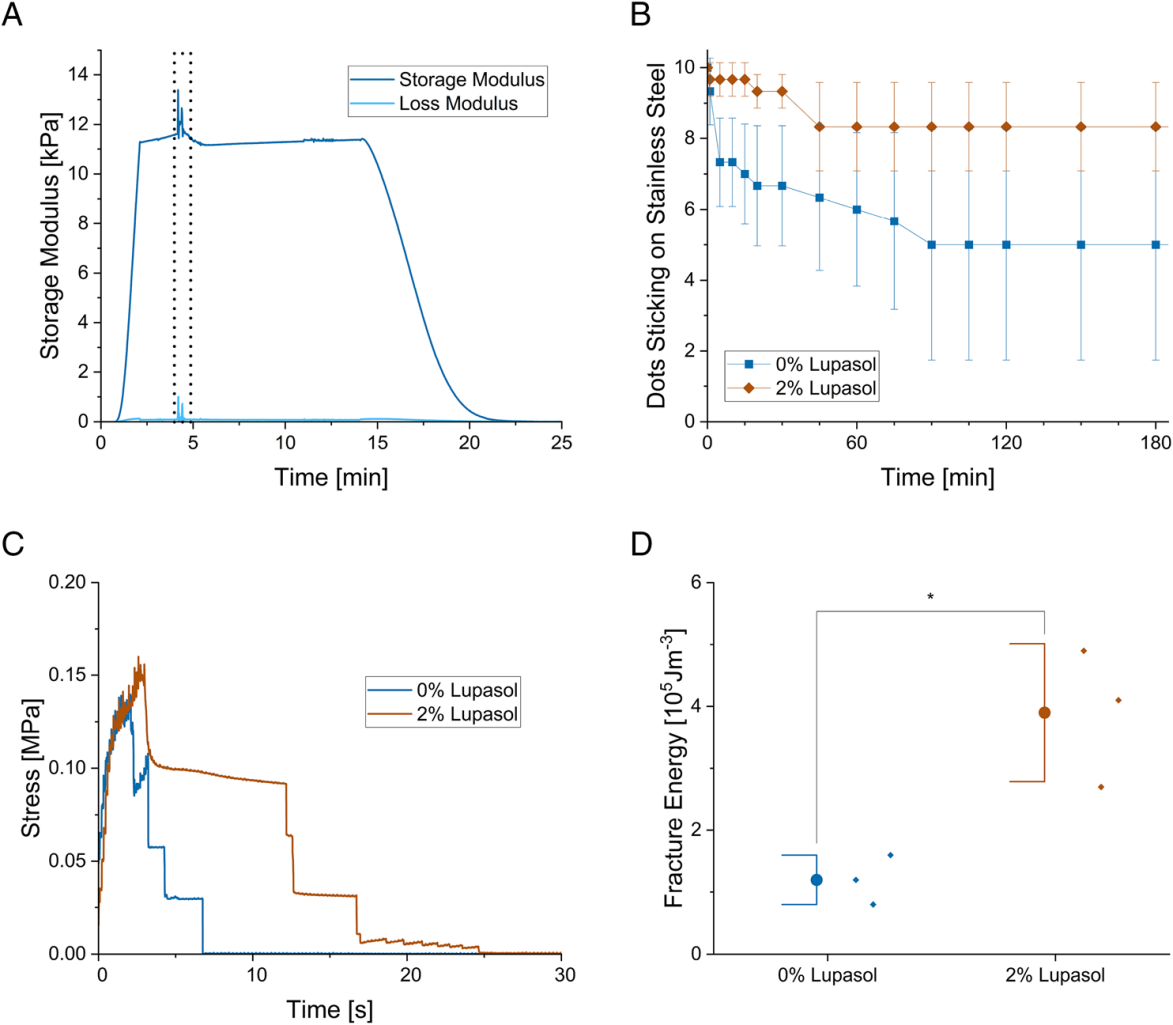
Rheology and improved adhesion of films. (A) Storage modulus of one sample without Lupasol during curing (between 1 and 3 min) and cleaving (starting at 15 min). Irregularities were introduced by adding dH_2_O around the sample to prevent shrinking due to drying (marked with a dotted box). (B) Statistics of adhesion of 10 drops of 2 µL of formulation with and without Lupasol WF cured on stainless steel. After immersion in water at room temperature, the sticking dots were counted between 0 and 180 min, showing that Lupasol WF increases the adhesion of the film, with the error bars showing the standard deviation between the three samples. A pull test on the rheometer led to the following data: C) Average pull force over time for formulations with and without Lupasol WF. (D) Fracture energy of the two formulations (adhesive failure at the quartz interface for the formulation without Lupasol, cohesive failure within the formulation with 2% Lupasol).

### 3.2 Lupasol WF Improved Adhesion of Photoreversible Films

While the viscosity and cleavage kinetics of PEGdiPDA‐based networks were suitable for the target application, the adhesion of the base formulation was not sufficient for downstream processes. In general, coating of industrial materials, such as untreated metal or glass surfaces, with aqueous polymer film formulations is a challenge due to their typically low and unsteady surface adhesion [62]. While surface pre‐treatments can be made on glass or silicon wafers by condensation (silanization) [63] or self‐assembly [64], further options exist for the treatment of metals, metal oxides, and semiconductors via self‐assembling layers [65, 66]; however, these approaches introduce additional processing steps and are often more challenging for geometrically complex substrates. In this context, additional strategies have been explored to enhance polymer film adhesion without surface pre‐treatment, including hydrogen bonding [67], engineering network topology [68], incorporating microcrystalline cellulose and copper chloride for copper coordination [69], and employing nanoparticles [51, 70].

In our formulations, several common adhesion promoters, such as silane‐acrylates (e.g. 3‐(Trimethoxysilyl)propyl acrylate), dopamine, or phosphonates (e.g. 11‐Phosponoundecyl acrylate), did not improve adhesion on metallic surfaces (Figure S4). However, the polyethyleneimine‐based adhesion promoter Lupasol WF (BASF) significantly increased the adhesion of photodegradable coatings on stainless steel (Figure S1). While this addressed a main challenge in applying these reversible protective films in industrial applications, it was critical to confirm that the inclusion of Lupasol WF in the formulation did not impair film formation and degradation. For immersion‐based treatments, such as electroplating or browning, the coating should remain adhered during liquid immersion. We cast ten drops of the formulation cured on stainless steel and immersed them in water at room‐temperature (Figure S5). Lupasol WF improved the adhesion of the reversible films for up to 45 min. For longer time scales, the adhesion variability increased, however, the data still indicate adhesion with Lupasol up to 180 min, as demonstrated by the increased resistance to debonding of the photocured material when submerged in water (Figure 2B).

To quantify the adhesive performance of the films and the effect of Lupasol WF in detail, the two formulations (containing 0 wt.% or 2 wt.% Lupasol WF) were characterized by utilizing the in‐situ curing and pull‐out tests in the rheometer. This way, high strain precision was achieved while minimizing the material consumption as well as measuring the shrinkage prior to the pull‐out (Figures S6 and S7). The pull‐out test characterized the films in their as‐formed state without potential swelling through prolonged exposure to water (Figure 2C). The strain at break values were 33 ± 17% and 36 ± 2% for the tested triplicates of PEGdiPDA with and without 2 wt.% Lupasol, respectively (Figure S8, Table 1). In addition, the maximum pull forces needed for failure did not change significantly between samples with (8.33 ± 2.55 N) and without (8.98 ± 2.24 N) 2 wt.% Lupasol WF. However, the fracture energy was 3.25‐fold larger for the formulations with 2 wt.% Lupasol WF (3.9 ± 0.9 10^5^ J m^−3^) than without (1.2 ± 0.3 10^5^ J m^−3^; Figure 2D), although Lupasol lowers the storage modulus of the material significantly as shown below. In addition, qualitative comparison of the strain modulus agrees with the rheometer measurements, indicative of a decreased modulus for the sample with Lupasol that could be explained by the formation of aza‐Michael bonds between amines on the Lupasol and acrylates in the network precursors [71, 72].

**TABLE 1:** Summary of adhesion analysis of PEGdiPDA/Lupasol WF films. Plate material given in adhesive failure mode indicates on which plate the gel delaminated during the pull‐out test, here the bottom quartz plate.

Lupasol WF weight concentration (wt.%)	Strain at break (%)	Maximum Force (N)	Failure Mode	Fracture energy (x10^5^ J m^−3^)
0	36 ± 2	8.98 ± 2.24	3 adhesive (quartz)	1.2 ± 0.3
2	33 ± 17	8.33 ± 2.55	3 cohesive	3.9 ± 0.9

The fracture energy of the films without Lupasol was similar to the previously reported fracture energy of polyacrylamide hydrogels determined by lap shear tests (1.2 × 10^5^ J m^−3^) [73]. Nevertheless, it should be mentioned that the rheometer‐based testing methodology is prone to a small overestimation of data if the cleaning of the resin neighboring the test area is done imperfectly [74], which applies for all other plate–plate rheometer tests.

The similar maximum force yet higher fracture energy for the films with adhesion promoter was ascribed to the difficulty to simultaneously achieving high adhesion strength (i.e., focusing on interfacial toughness) and high bulk energy dissipation strategies for improving adhesion. Among the two strategies, it is known that bulk energy dissipation contributes more to the adhesion energy than the adhesion strength in adhesive elastomers [67]. The improvement in adhesion was ascribed to the branched PEI having polar amino groups [75], high cationic charge density, strong Van der Waals or ionic/polar group interactions promoting interfacial adhesion with metals, charged polyelectrolytes, and cells [76]. The robust adhesion of PEI on metals has been reported for gold [76], iron [77], and stainless steel [78] substrates, as well as heavy metal ions [75, 77]. The free electron pair on the nitrogen allows PEI to act as a nucleophilic agent and to establish coordination bonds with metals, forming complexes [77]. Importantly, the mode of failure also changed from adhesive, with detachment from the quartz plate for all samples with 0 wt.% Lupasol WF, to cohesive for samples with 2 wt.% Lupasol WF. This indicated that the adhesion to both quartz and aluminum was larger than the maximum normal force the film could withstand before failing, qualitatively proving the positive change in adhesive performance. These findings underline the improved adhesion achieved by the addition of Lupasol WF on a variety of materials, from stainless steel to quartz glass.

### 3.3 Photoreversible Adherent Films

To exclude potential counterproductive interactions between Lupasol WF and the base polymer networks, we investigated the photophysical properties of the formulations. We analyzed the UV–vis spectra of dilute formulations (1:80 in water) with and without Lupasol WF prior to simulated curing, after simulated curing (λ_1_ = 405 nm; t = 2 min), and after photodegradation (λ_2_ = 365 nm; t = 30 min). There was no apparent change in the UV–vis spectra upon inclusion of Lupasol WF and upon simulated photocuring. While no conclusions can be drawn from this finding about the curing of the network, no significant additional absorption in the region around 365 nm was introduced that could hinder the cleavage. In formulations with and without Lupasol WF, a redshift in the absorption in the UV and near‐UV range occurred upon photodegradation, which is typical for photocleaved oNB‐compounds [38, 39, 79]. For both formulations, the absorption from before to after cleavage undergoes the same characteristic change, flattening out the absorption between 300 and 400 nm wavelengths (Figure 3A). Also, comparing the FT‐IR spectrum of both formulations, no significant difference was observed before or after cleavage (Figure S9) between the formulations with and without Lupasol WF, which would become apparent if the adhesion promotor would be involved in chemical interactions with the network precursors or the degradation process of the formed networks. On a molecular level, these two findings suggest that the addition of Lupasol WF should not hinder the ability of the PEGdiPDA cross‐linker to cleave and hence of the full network to degrade.

**FIGURE 3:**
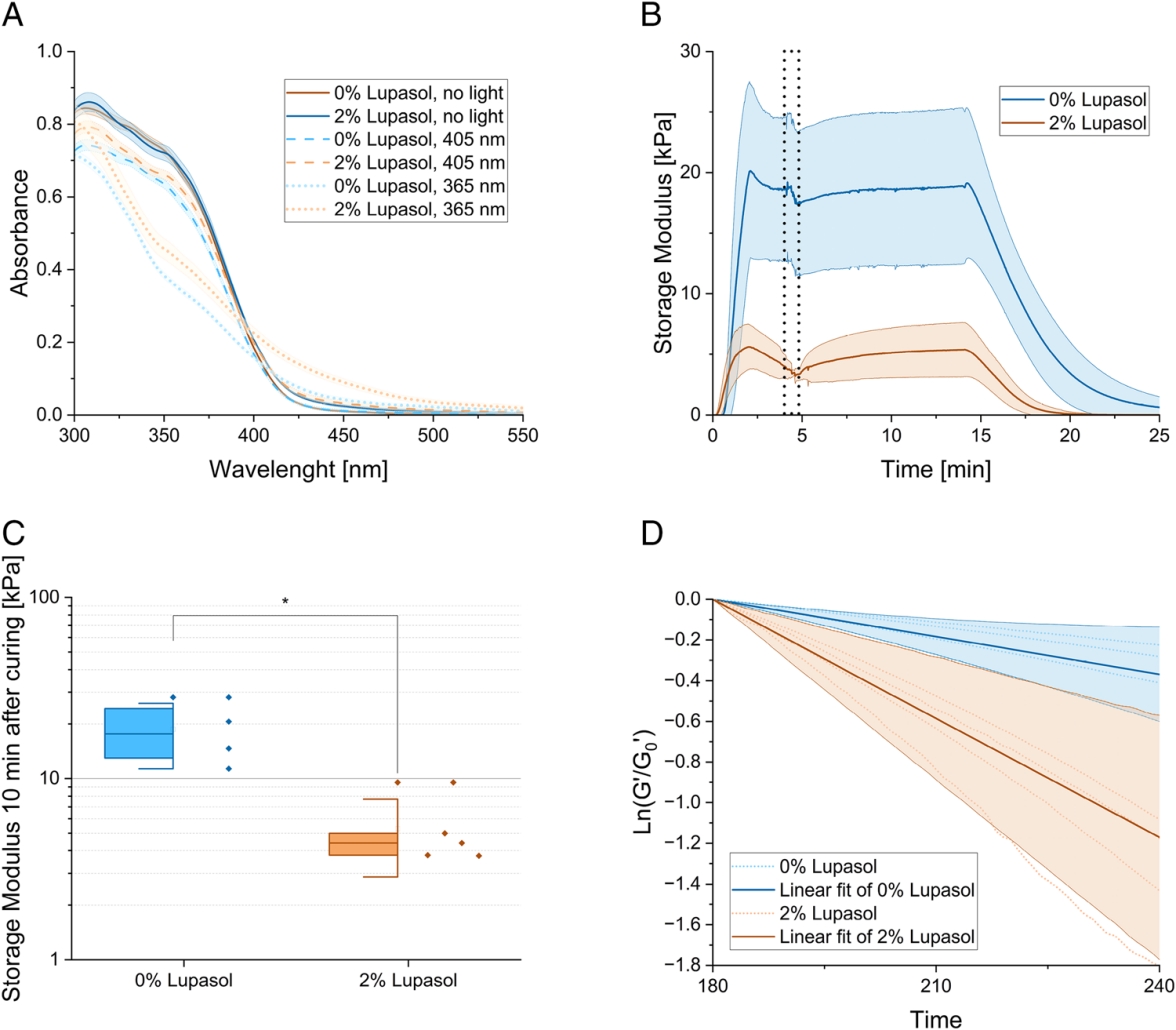
Adhesion promoter improves cleavage behavior. (A) Absorption normalized to a pathlength of 1 cm of material before irradiation as well as after curing at 405 nm and cleaving at 365 nm light. (B) Typical curves of storage and loss modulus for formulations with 0% and 2% Lupasol. The material is cured with 405 nm light for 2 min. After 4 min, dH_2_O is added around the sample to prevent drying, leading to a temporary disturbance of the measurement (marked with a dotted box). The light at 365 nm was turned on at 15 min after the plateau stabilized and induced photodegradation of the material. (C) Plateau modulus 10 min after curing films with 0% and 2% Lupasol. The difference between the two formulations is statistically significant (*p* ≤ 0.05). D) Exponential fit of the shear modulus to derive the effective rate constant *k* for the two formulations with and without Lupasol.

However, the most important aspect for industrial application is the behavior of the bulk material—the maximal modulus reached as well as the rate and degree of cleavage. As measured via shear rheology, the average curves of storage and loss modulus during curing and cleaving show a typical behavior for both formulations. After curing with 405 nm light for 2 min, the films reached a stable plateau modulus and, upon irradiation with 365 nm cleaved with the characteristic exponential decrease in storage modulus (Figure 3B). To compare the two formulations, the storage modulus 10 min after curing was measured (Figure 3C). While the formulation with 0% Lupasol WF exhibited a plateau modulus of 18.7 ± 6.4 kPa, the formulation with 2% Lupasol WF had a plateau modulus of 5.3 ± 2.2 kPa. This indicates that Lupasol WF may interfere with network formation; however, the formulations formed networks in both cases. This could be due to the large size of the polyethyleneimines in Lupasol WF (*M_n_* ∼25 000 g mol^−1^) compared with PEGdiPDA (*M_n_* ∼4200 g mol^−1^) and PEGA (*M_n_* ∼480 g mol^−1^). Such large molecules may disrupt the regular network structure, impacting its mechanical elasticity. Another possible explanation could be the formation of bonds through aza‐Michael addition between the primary amines of Lupasol WF and acrylates [71, 72]. This process, however, likely occurs at longer timescales than the curing of the material [71], which should minimize the influence of the aza‐Michael addition during curing. The presence of some aza‐Michael bonds could alter the network structure and thereby explain the decrease in observed modulus; however, the materials degraded fully in the presence of Lupasol WF, suggesting that any non‐photocleavable, aza‐Michael bonds (if present) did not impede photodegradation.

Having shown that Lupasol WF did not prohibit network formation and that it improved surface adhesion, we investigated whether the photodegradation of the films was preserved. The exponential decrease in modulus of the photocleavage can be used to quantify the effective rate constant, *k* [45, 58, 60]. For the calculation of the rate constant, a time frame of 1 min was chosen where all measured samples were in the linear range after the start of the irradiation with 365 nm light. For the samples without Lupasol WF, *k* = ‐0.00613 ± 0.00014 s^−1^ and for the gel with 2% k equals ‐0.01952 ± 0.00039 s^−1^ (Figure 3D) While there is a clear difference between the two formulations, both effective rate constants were within the reported range for similar PEGdiPDA‐based networks. This confirmed that the inclusion of Lupasol WF did not impede the ability of the films to form and photodegrade. While the current data does not allow a definite conclusion on the reason for the variation in cleavage rate, this may be caused by changes in the network structure, e.g., network connectivity [58] or mass fraction of polymer in the backbone chain [80], upon addition of Lupasol. In addition, local pH‐dependent shifts might have an influence on the rate of oNB cleavage [81].

In total, the rheological evaluation showed that the addition of Lupasol WF did not compromise the photodegradation of the films; in fact, the rate of cleavage increased slightly for the samples with 2 wt.% Lupasol WF compared with the samples without. On the other hand, the plateau storage modulus was reduced significantly by the addition of Lupasol WF; however, the mechanical properties remain sufficient for many coating applications, and the properties of the film can be adjusted by the total weight percentage of the polymers in the formulation or the length of PEG‐chains used in the synthesis of PEGdiPDA or the PEGA [82].

### 3.4 Digital Printing of Films

Novel digital printing technologies offer a wide range of benefits that are attractive for small‐scale applications, such as the watch industry with small product batches and high demand in resolution and quality. For such applications, the decreased speed compared to roll‐to‐roll (R2R) techniques, such as gravure printing or screen printing, is compensated by the high flexibility and the ability to print on three‐dimensional surfaces of the aerosol jet printing technology.

Aerosol jet printing has some specific requirements for the used inks, where our low‐viscosity, aqueous formulations offer a convenient solution. The ink viscosity needs to be in the range of a few cP for ultrasonic atomization, and a combination of low‐ and high‐boiling‐point solvents is beneficial for the printing process [83]. The formulation with Lupasol WF was diluted from 15 wt.% polymer content to 1.67 wt.% with IPA, n‐Butanol and dH_2_O, according to the protocol described in the materials and methods section. This formulation allows not only a mix between solvents with high (n‐Butanol) and low boiling points (dH_2_O, IPA), but it also lowers the surface tension compared to a pure water‐based formulation. The latter is especially important for printing on low‐energy surfaces, as an ink with high surface tension is prone to dewetting and formation of droplets.

To demonstrate the printability of the protective films, the formulated ink was printed on three different materials: On glass, analyzing the resolution and thickness on a smooth surface; on stainless steel to show the printing on a standard, brushed surface; and on copper to demonstrate the temporary surface protection by the film. A single line was printed on glass with a mean linewidth of 28.5 µm and a mean height of 0.94 µm (Figure 4A, Figure S10) as well as on stainless steel with a mean linewidth of 31.9 µm and a mean height of 0.53 µm (Figure 4B). The pronounced surface roughness of brushed stainless steel makes the exact measurement of the film thickness difficult, as the surface of both film and substrate were not uniform. To ensure a robust layer, the printing parameters were adjusted to a higher ink deposition when printing patches (1 × 2 mm), This resulted in thinner patches on stainless steel, with a height of 1.93 µm (Figure 4C) compared with 4.31 µm on glass.

**FIGURE 4:**
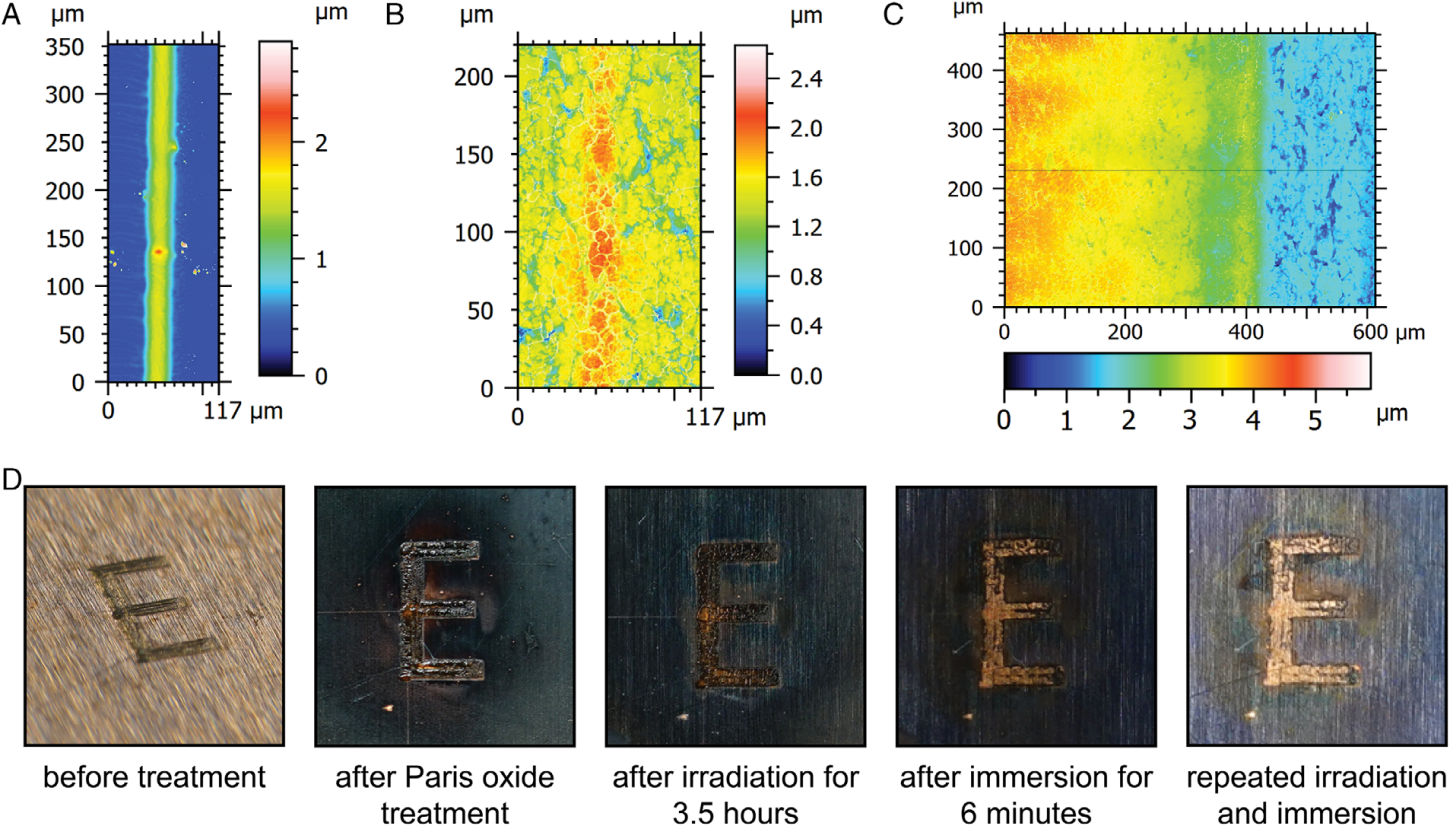
Digital printing and use of protective coating. (A) Topography of a line printed on glass and (B) on brushed stainless steel. (C) Border of a patch printed on stainless steel. (D) Photographic image of the letter “E” (1.25 × 2.5 mm) printed with the formulation containing Lupasol WF on brushed copper, treated with Paris oxide, irradiated under 365 nm UV‐light for 3.5 h, immersed in water for 6 min. For the last state, sample was again irradiated for 10 min and immersed in water for 1 min to remove residuals.

Toward industrial application, the protective capability of the materials was shown on copper. The application of the coloring agent “Paris oxide,” commonly used for blackening copper or silver in decorative applications, was used as a demonstrator. When Paris oxide is in contact with copper, the surface quickly changes color to a bluish hue of black while the solvents of the Paris oxide evaporate. The letter “E” (1.25 × 2.5 mm) was printed and cured on brushed copper. Then Paris oxide was applied and the film irradiated for 3.5 h with light at 365 nm wavelength to ensure sufficient degradation of the protective film, resulting in a weakened coating that detaches after immersion in water for 6 min. Repeated irradiation and immersion further reduce residuals of the coating.

This first demonstrator for using PEGdiPDA‐based coatings for temporary surface protection shows a visible reproduction of the printed shape by the untreated surface and opens up a field for further experimentation targeted for the needs of specific surface treatments. These future opportunities will allow for additional optimization of printing path and parameters. In addition, depending on the targeted application, further investigations are needed to tailor the formulation properties (e.g., mechanics in the printed state) for specific applications by varying the polymer, water, and/or solvent contents. In the present work, we were not able to quantify the modulus of the printed structures, and future work should focus on characterizing these properties and relating them to formulation parameters.

## 4 Conclusions

The combination of a photoreversible, PEGdiPDA‐based network with Lupasol WF formed a material that allowed the successful implementation of a photoreversible and printable protective film. In addition, the inclusion of Lupasol improved adhesion on a variety of untreated surfaces compared with the base material. Notably, the fracture energy of the PEGdiPDA films more than tripled and their failure mode became cohesive when the adhesion promoter Lupasol WF was included. The rheometer‐enabled adhesion testing methodology involving in situ curing and pull‐out is well suited for adhesion characterization of small amounts of photocurable soft materials. The photodegradation of the network was preserved when Lupasol WF was present, allowing light‐responsive removal of protective coatings. We further demonstrated that the material is printable with aerosol jet printers as a state‐of‐the‐art digital printing technique, where prints of an industry‐relevant resolution can be manufactured on different surfaces, such as glass, stainless steel, or copper. In a first demonstrator, the film adhered to copper during blackening of the surface, protected the print area from the treatment, and dissolved upon irradiation with light at a wavelength of 365 nm and easy removal in water. This makes the material interesting as a platform technology that can be adapted to the specific needs of an application through modification of the formulation, film thickness, and process parameters of treatment and cleavage.

## Funding

This work was made possible thanks to the funding provided by the Strategic Focus Area Advanced Manufacturing (SFA‐AM) of the ETH Domain Switzerland for the project DiPrintProtect.

## Conflicts of Interest

The authors declare no conflicts of interest.

## Supporting information




**Supporting File**: marc70408‐sup‐0001‐SuppMat.pdf.

## Data Availability

The data that supports the findings of this study are available in the supplementary material of this article.
