# Supplementary material for: Digital Printing of Photoreversible Protective Films
Source: Macromol Rapid Commun. 2026 Aug 17;47(20):e70408. doi: 10.1002/marc.70408 (PMC13681341; doi:10.1002/marc.70408)
Supplement: Supplementary file 1 [file MARC-47-e70408-s001.pdf]

Supporting Information

Digital Printing of Photoreversible Protective Films

Morris Wolf[1], Alper Balkan[2], René Schneider[3], Stefan Mommer[1], Yves Leterrier[2], Mark W. Tibbitt[1]

1 Macromolecular Engineering Laboratory, ETH Zurich, CH-8092 Zürich, Switzerland
2 Laboratory for Processing of Advanced Composites (LPAC), École Polytechnique Fédérale de Lausanne (EPFL), CH-1015 Lausanne, Switzerland
3 Laboratory for Functional Polymers, Swiss Federal Laboratories for Materials Science and Technology (Empa), CH-8600 Dübendorf, Switzerland

Contents

Figure S1: ^{1}H-NMR of the synthesized crosslinker PEGdiPDA, recorded in DMSO-d_6
Figure S2: GPC measurement of unfunctionalized NH_2-PEG-NH_2 and synthesized PEGdiPDA
Figure S3: UV-Vis spectra of the GPC measurement.
Figure S4: Performance of various adhesions promoters on brass as additives
Figure S5: Photograph of 2% Lupasol WF formulation cured and immersed in water
Figure S6: In-situ curing and pull-out tests for PEGdiPDA films without Lupasol WF
Figure S7: In-situ curing and pull-out tests for PEGdiPDA films with 2 wt% Lupasol WF
Figure S8: Strain at break of the two formulations during the pull test on the rheometer
Figure S9: FT-IR curves of formulations with 0 and 2 wt%
Figure S10: Topography in 3D of a line printed on glass.
References

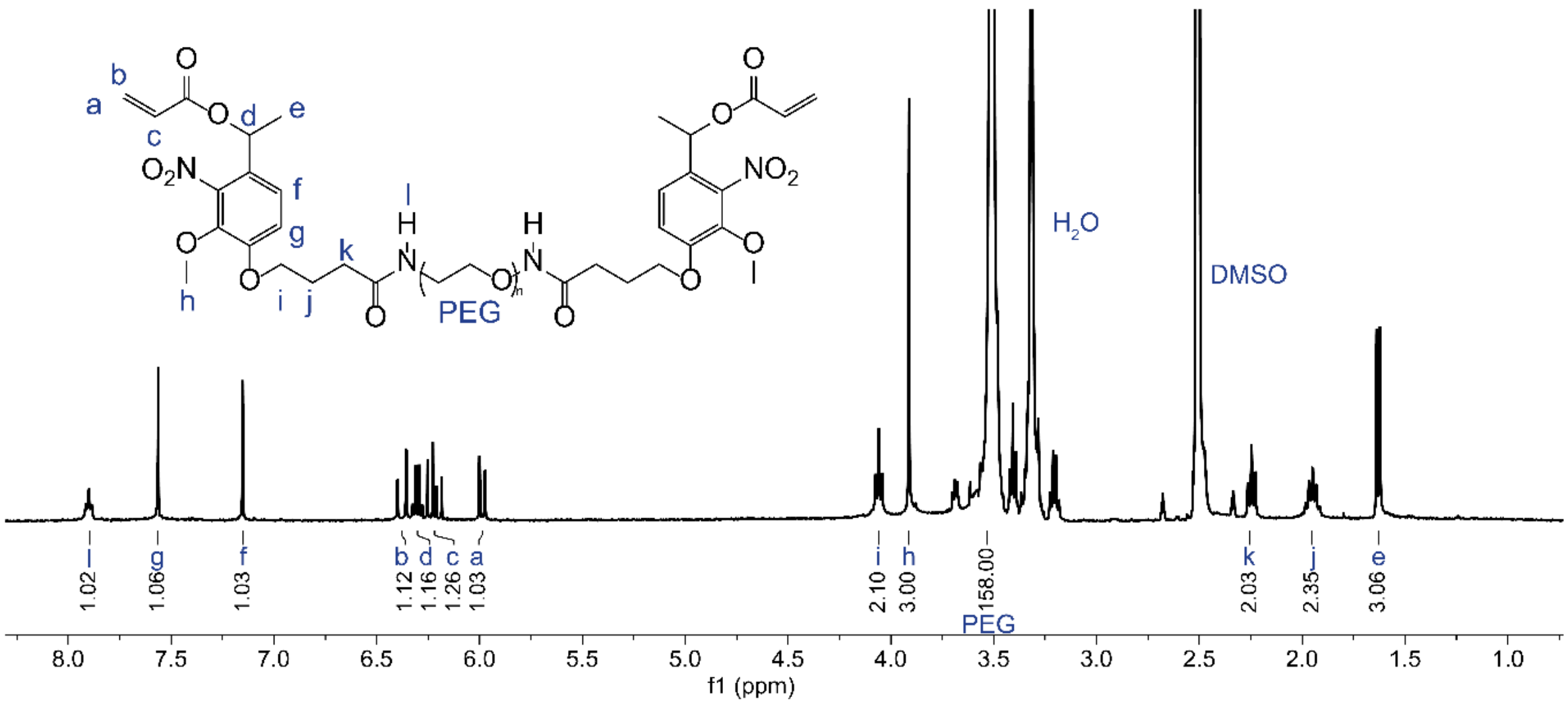

Figure S1: ^{1}H-NMR of the synthesized crosslinker PEGdiPDA, recorded in DMSO-d_6. Integrals are normalized to one side of the molecule for reference to the number of hydrogens on the nitrobenzyl ether moiety.

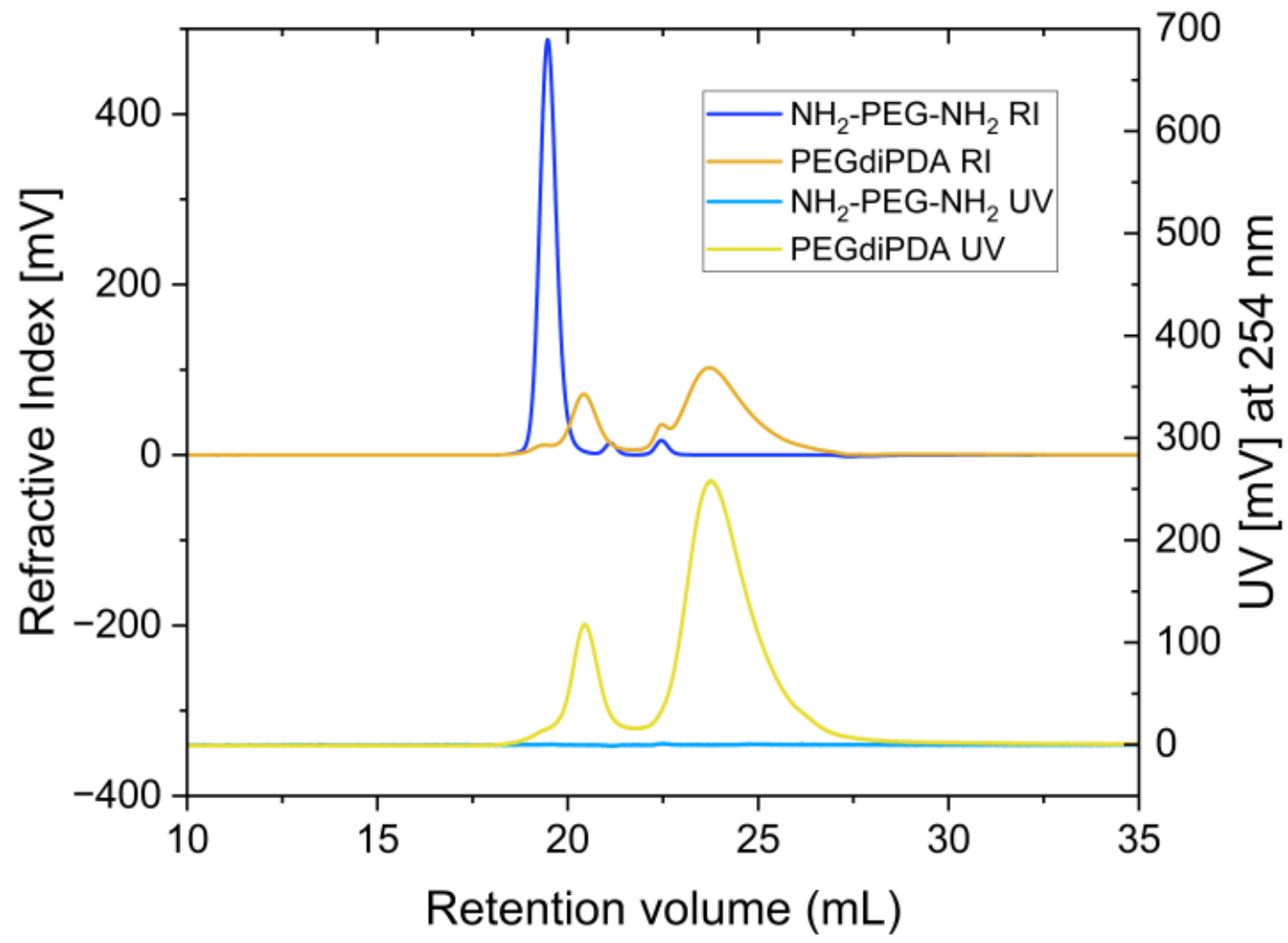

Figure S2: GPC measurement of the unfunctionalized NH_2-PEG-NH_2 as well as the synthesized PEGdiPDA. In the refractive index, a clear shift is visible, from the unfunctionalized PEG to higher retention volumes with functionalization. Besides a small fraction of monofunctional PEGPDA at 20.35 mL, the main PEGdiPDA shows a signal at 23.70 mL. As opposed to the NH_2-PEG-NH_2, both PDA-related peaks are UV-absorbing, proving the functionalization with the photoactive residue. With increasing PDA functionalization, a shift to higher retention volumes is observed, which can be explained through the addition of the highly hydrophobic PDA residue to the PEG chain, resulting in smaller hydrodynamic radii. The UV-VIS data supports this peak assignment, showing higher absorption intensities for the difunctional over monofunctional species, since the UV-absorbing groups are attached to the non-absorbing NH_2-PEG-NH_2.

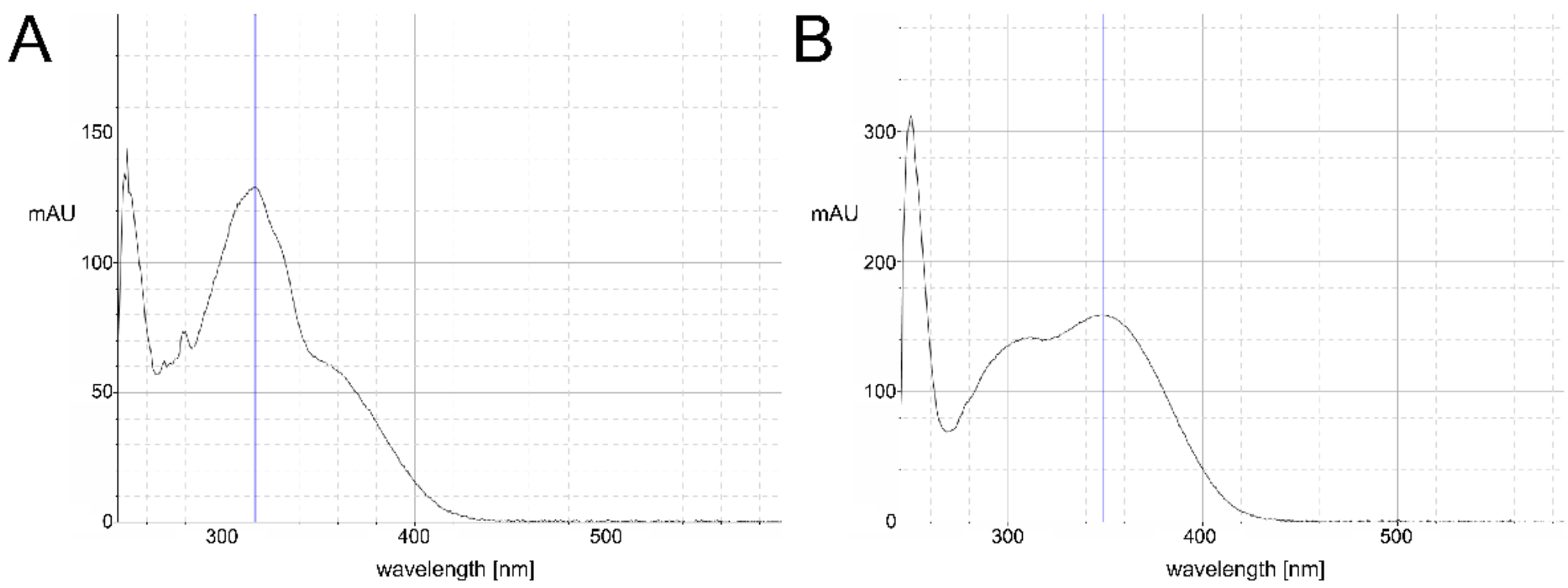

Figure S3: UV-Vis spectra of each individual peak at a retention volume of VR = 20.35 and 23.70 mL of the GPC elugram, showing a clear increase in absorption intensity from the monofunctional derivative (A) to the bifunctional PEGdiPDA (B).

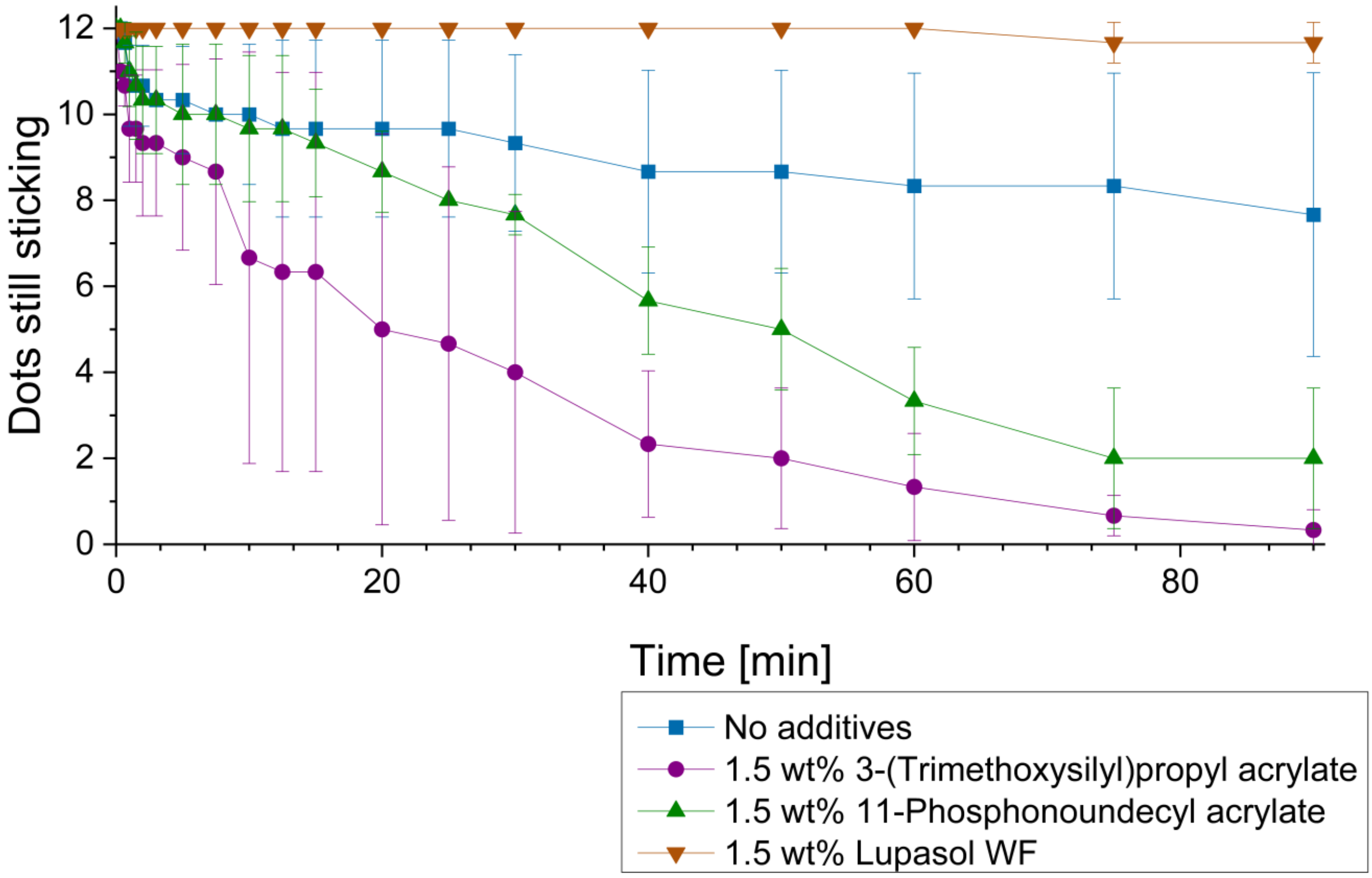

Figure S4: Performance of various adhesions promoters on brass as additives to a formulation with 8.2 wt% PEG-diacrylate (PEGDA, molecular weight 4000, Sigma-Aldrich), 6.8 wt% PEGA, 0.5 wt% LAP and 1.5 wt% of adhesion promoter. 12 drops of 5 µL were pipetted for each formulation, cured under 405 nm light for 7 minutes at 14.2 mW/cm^2 and immersed in water. The graph shows the drops still sticking after a certain time immersed. The experiment was repeated 3 times for every formulation.

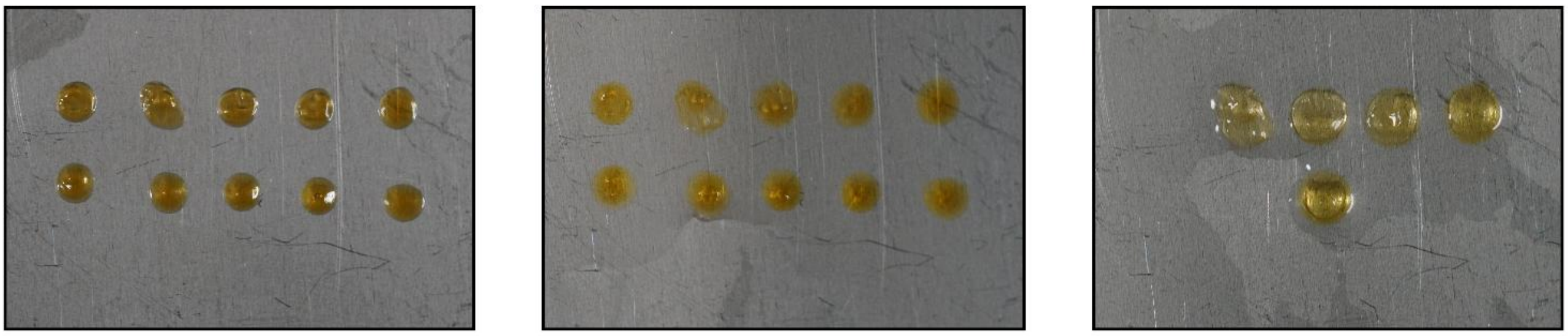

cured 2 min 75 min

Figure S5: Photograph of 10 dots (2 µL) of the 2% Lupasol WF formulation cured on a stainless-steel plate on the left and the same dots after 75 min in water on the right, where 5 dots are still sticking as an example of the immersion-based adhesion tests.

In situ curing and pull-out tests of PEGdiPDA films without and with Lupasol WF

The adhesion tests for PEGdiPDA gels without and with Lupasol are displayed in **Figures S3** and **S4** respectively. There was 0–3 µm gap reduction (initial gap: 20 µm) as well as a force build-up following the initial 240 s prior to the pull-out. These were ascribed to the combined drying and UV-curing of the gels. The serrated force profiles during the pull-out stage (**Figure S2b**) indicated strain hardening, potentially accompanied with micro-scale deformations taking place in the network structure, i.e., micro-fractures at the load-bearing junctions of the network.[1] Non-linear mechanical response in gels were previously also associated with topological defects.[2] Here it is worth mentioning that the polyacrylate PEGA forms coil-like structures in water between PEGdiPDA molecules,[3] which could also be responsible for the serrated force profile as these coils could be expected to extend or break upon pulling. The force profiles appeared less serrated for the profiles of PEGdiPDA with 2 wt.% Lupasol WF (**Figure S3**), suggesting the participation of Lupasol WF to the overall mechanical response of the material and allowing less intense strain hardening structural effects. The fracture of gels both without and with Lupasol WF occurred mostly in 2 steps, and for measurement 1 without Lupasol WF, this was accompanied with jumps in displacement (i.e., non-gradual increase). This becomes more apparent at the stress–strain profile in **Figure S3c**. The blue regions indicating that jump, that could cause an inflated area, were excluded from fracture energy calculations and could be interpreted as artifacts stemming from releases of stored elastic energy in a catapult-like moving manner of the rheometer plate.

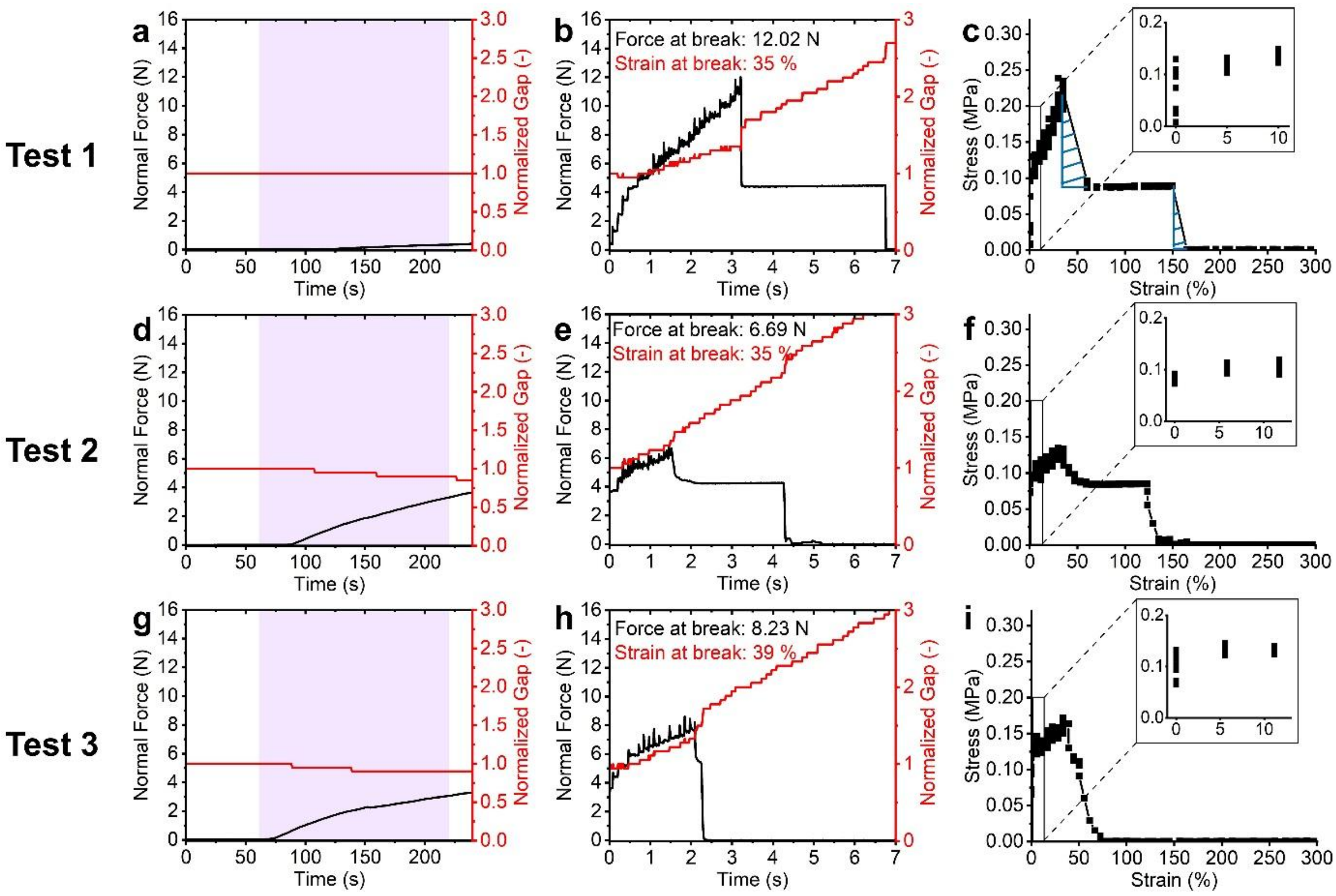

Figure S6: In-situ curing and pull-out tests for PEGdiPDA films without Lupasol WF: (a,d,g) photo-curing of the gels where purple region indicates the photo-curing duration, (b,e,h) pull-out tests showing normal force and re-normalized gap change by time at a pull speed of 5 µm s^{-1}, (c,f,i) stress - strain profiles with blue regions indicating the omitted area for energy calculations (blue regions only in c due to non-continuous strain progression).

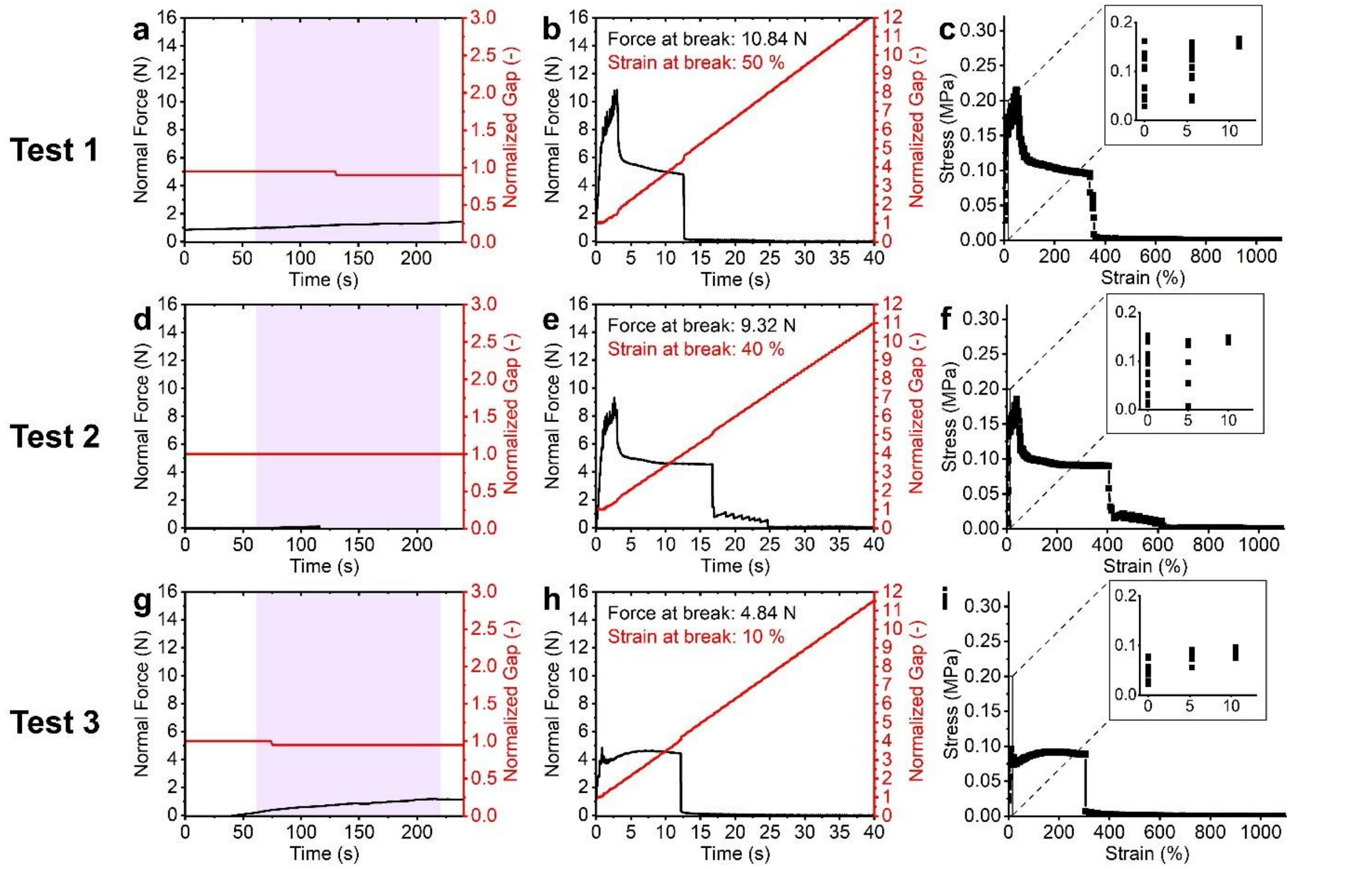

Figure S7: In-situ curing and pull-out tests for PEGdiPDA films with 2 wt% Lupasol WF: (a,d,g) photo-curing of the gels where purple region indicates the photo-curing duration, (b,e,h) pull-out tests showing normal force and re-normalized gap change by time at a pull speed of 5 µm s^{-1}, (c,f,i) stress–strain profiles.

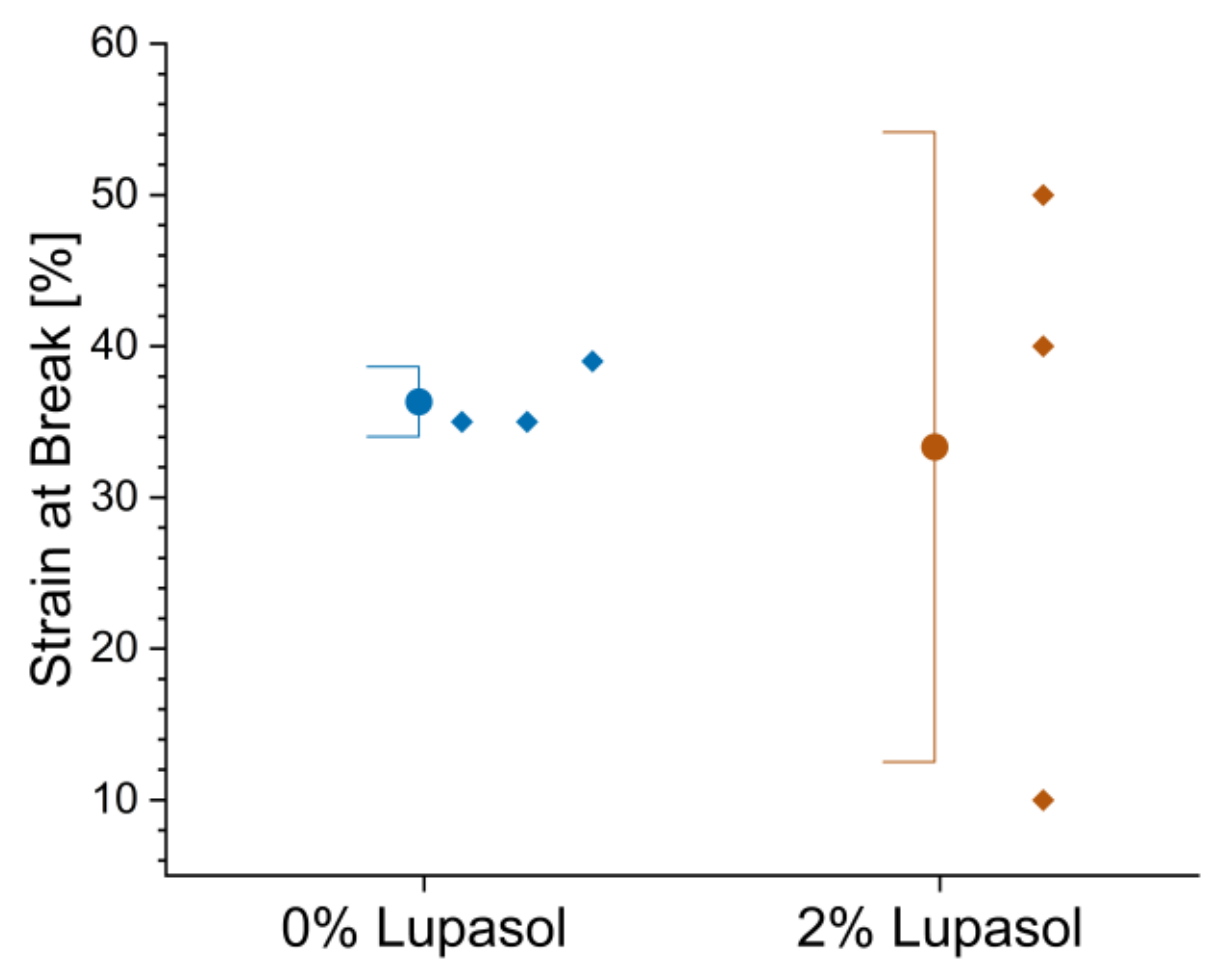

Figure S8: Strain at break of the two formulations during the pull test on the rheometer

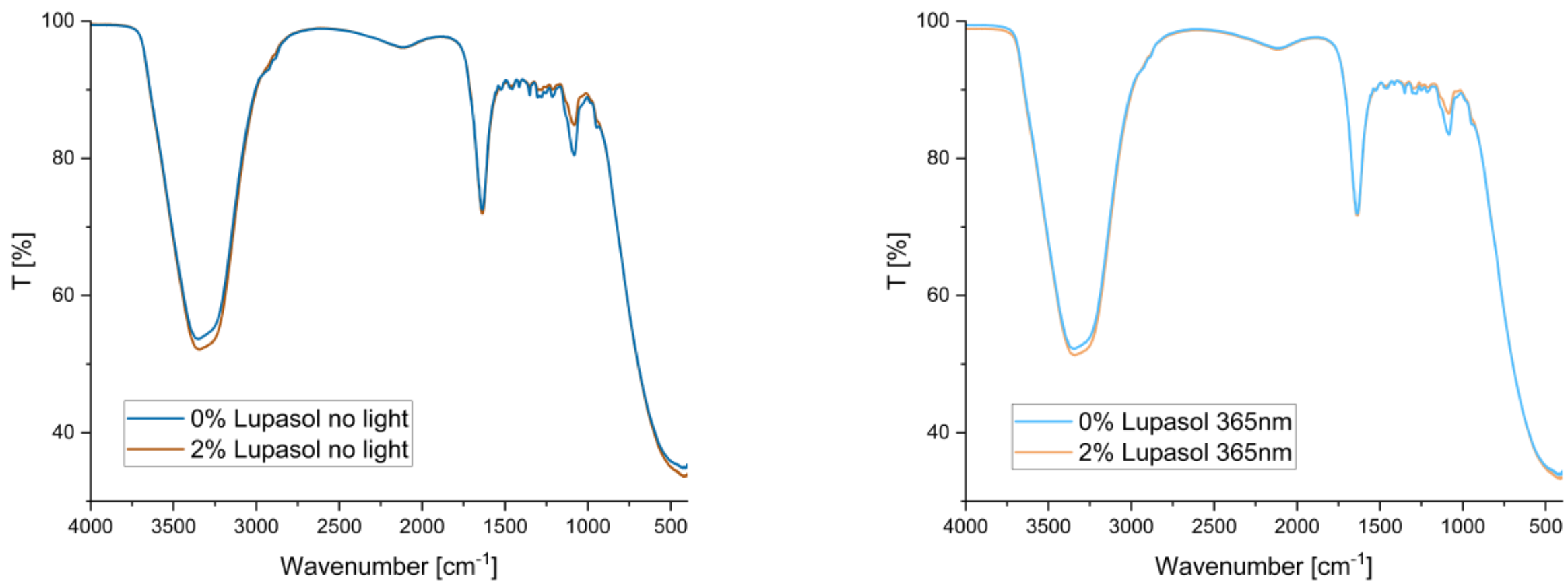

Figure S9: FT-IR curves of formulations with 0 and 2 wt% Lupasol before irradiation and after curing with 405 nm and cleaving with 365 nm light.

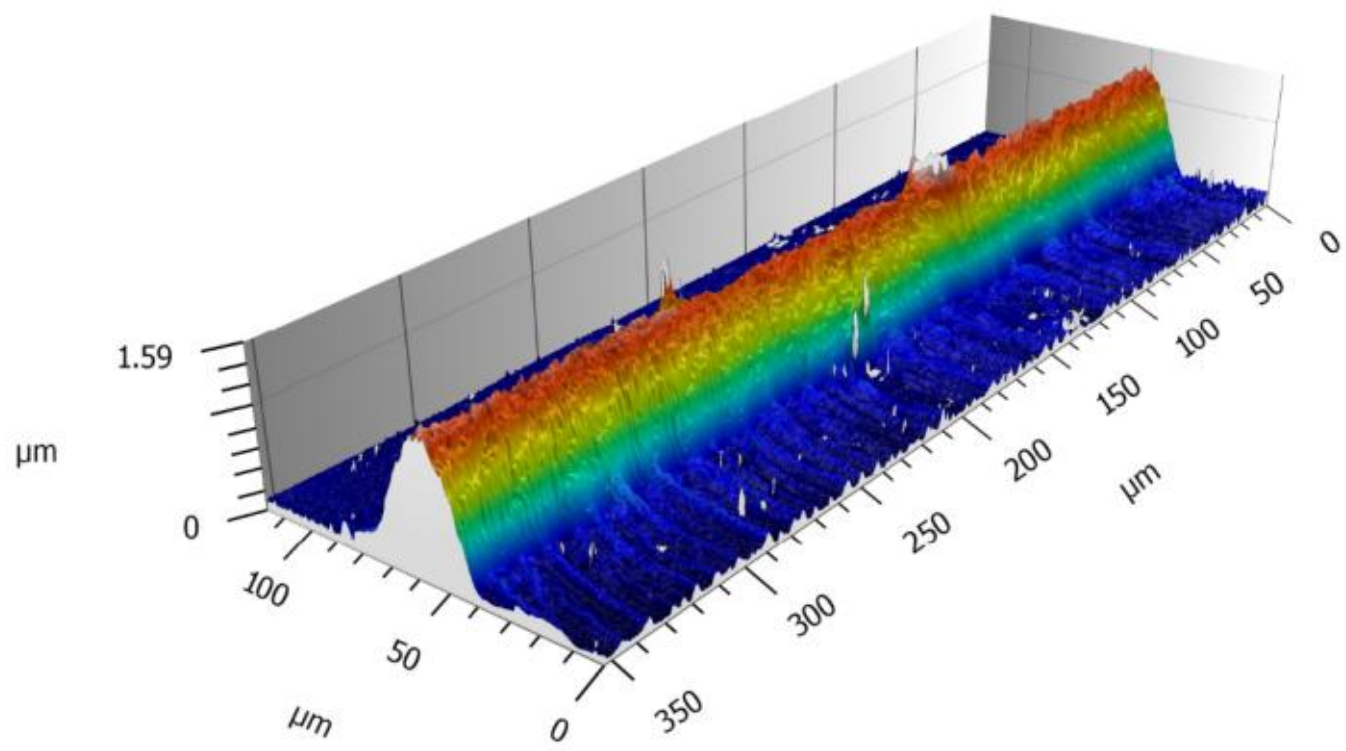

Figure S10: Topography in 3D of a line printed on glass.

References:

(1) Burla, F.; Dussi, S.; Martinez-Torres, C.; Tauber, J.; van der Gucht, J.; Koenderink, G. H. Connectivity and Plasticity Determine Collagen Network Fracture. *Proceedings of the National Academy of Sciences* **2020**, *117* (15), 8326–8334. https://doi.org/10.1073/pnas.1920062117.

(2) Bouzid, M.; Del Gado, E. Network Topology in Soft Gels: Hardening and Softening Materials. *Langmuir* **2018**, *34* (3), 773–781. https://doi.org/10.1021/acs.langmuir.7b02944.

(3) Kloxin, A. M.; Tibbitt, M. W.; Anseth, K. S. Synthesis of Photodegradable Hydrogels as Dynamically Tunable Cell Culture Platforms. *Nat Protoc* **2010**, *5* (12), 1867–1887. https://doi.org/10.1038/nprot.2010.139.
